# “An Intrinsic Program Determines Key Age-Associated Changes in Adaptive Immunity That Limit Response to Non-Pathogens”

**DOI:** 10.3389/fragi.2021.701900

**Published:** 2021-06-24

**Authors:** Susan L. Swain, Olivia Kugler-Umana, Susan L. Tonkonogy

**Affiliations:** ^1^ Department of Pathology, University of Massachusetts Medical School, Worcester, MA, United States; ^2^ College of Veterinary Medicine, North Carolina State University, Raleigh, NC, United States

**Keywords:** age-associated B cells, aging, T cells, adaptive immunity, B cells, germ-free mice

## Abstract

As mice age their adaptive immune system changes dramatically, leading to weakened responses to newly encountered antigens and poor efficacy of vaccines. A shared pattern emerges in the aged, with both CD4 T and B cell responses requiring higher levels of pathogen recognition. Moreover, in aged germ-free mice we find accumulation of the same novel age-associated T and B cell subsets that we and others have previously identified using mice maintained in normal laboratory animal housing conditions, suggesting that their development follows an intrinsic program.

## Introduction

In aged individuals, influenza can lead to increased disability, frailty, and mortality. These have often been attributed to dysregulated immune responses and “inflammaging” (Swain and Blomberg, 2013; McElhaney et al., 2016; Nikolich-Žugich, 2018). Much of the reduced immunity is caused by a series of age-associated changes in naïve CD4 T and B cells, that are required to respond effectively to new antigens (Swain and Blomberg, 2013). Therefore, elderly individuals are highly vulnerable when new pathogens appear. Interestingly, many of the age-associated changes follow a predictable, highly reproducible course, though the mechanisms behind the changes are unclear (Swain and Blomberg, 2013). Interventions to improve vaccine efficacy for the aged (Haynes and Swain, 2006; Dorshkind and Swain, 2009; McElhaney et al., 2016; Nikolich-Žugich, 2018) require both better definition of the root causes of immune dysregulation and the detailed mechanisms responsible.

Here we present our perspective on the causes of age-associated changes, their downstream effects and how well-informed strategies might improve vaccines. Multiple components of the immune system are compromised with aging, but we focus on the adaptive responses that are most effective at eliminating pathogens. Much of our basic understanding comes from studies in murine model systems, with key aspects that were later confirmed in human studies when feasible. Here we focus on studies in mice. With age, many conventional naïve CD4 T cells and B cells are lost (Dorshkind and Swain, 2009; Lefebvre et al., 2016; Swain et al., 2017), causing restricted repertoires of receptors in both naïve T and naïve B cells (Dorshkind and Swain, 2009). We note that naïve CD4 T responses become more dependent on high levels of pathogen recognition (PR) signals (Jones et al., 2010; Brahmakshatriya et al., 2017), which limits responses except when there are ongoing infections. The responsive naïve B cells in aged mice are also highly dependent on PR (Hao et al., 2011; Rubtsov et al., 2011; Kugler-Umana et al., 2020). We suggest this is a desirable regulatory mechanism to dampen unnecessary immune responses and reduce autoimmunity. Thus, it would be risky to disrupt this program, but it may be possible to improve vaccines for older individuals by providing surrogates of pathogen infection.

### Age-Associated Changes in CD4 T Cell Response and T Cell Subsets

In the aged, there is a dramatic loss of naïve CD4 (and CD8) T cell number and repertoire due in part due to reduced production of naïve T cells. In response, the naïve T cell population decreases and cells with altered phenotype appear, perhaps driven by constitutive “homeostatic” cytokines that drive their homeostasis (Cicin-Sain et al., 2007; Kawabe et al., 2020).

We found aged naïve CD4 T cells express less of the pro-apoptotic protein Bim, which led to an increase in their lifespan compared to cells from young mice (Tsukamoto et al., 2009). With age, CD4 T cells lost function including proliferation, cytokine responses and expansion due to their increased cellular age (Tsukamoto et al., 2010). Supporting this, removal of existing peripheral CD4 T cells by depletion and their reconstitution thereafter, induced the generation of new naïve CD4 T cells in aged mice and these new cells had improved function (Haynes et al., 2005). Our studies stress that, when induced after infection or vaccination, many age-associated changes are both T cell intrinsic and independent of the aged host environment (Haynes et al., 2005; Tsukamoto et al., 2009; Tsukamoto et al., 2010; Devarajan et al., 2016). In contrast, memory CD4 T cells generated in early life, function well in aged mice, supporting the concept that naïve T cells are the most impaired (Devarajan et al., 2016) (Haynes et al., 2005). We also note that aging selectively reduces naïve CD4 helper function and generation of memory (Eaton et al., 2004; Lefebvre et al., 2016), which explains the poor response to vaccines for new pathogens (Brahmakshatriya et al., 2017).

We found that introducing toll like receptor (TLR)-activated dendritic cells (DC), as antigen-presenting cells (APC) to aged naïve CD4 cells, increased their response and this improved response was dependent on IL-6 production by the APC (Jones et al., 2010). We further analyzed the response of aged vs. young naïve CD4 T cells, expressing a transgenic T cell receptor (TCR) specific for influenza hemagglutinin (HA) by transferring them into host mice with an unrelated TCR transgene to focus on the aged naïve response. Hosts were primed intranasally with inactivated whole influenza virus. This allowed us to evaluate the direct impact of age on the donor T cell response to an influenza vaccine. Compared to young donor cells, the aged naïve CD4 T cells showed reduced expansion, reduced development of T follicular helper (TFH) cells and reduced germinal center (GC) TFH and CD4 memory. These in turn led to reduced GC B cell generation and influenza-specific IgG and B cell memory. When we introduced APC activated by stimulation with TLR agonists, each of the age-impacted responses were largely restored. We found aged naïve CD4 T cells respond poorly to IL-6, but the APC that were activated by pathogen recognition (PR) signals, produced high levels of IL-6 during cognate interaction, restoring the aged naïve CD4 T cell response (Brahmakshatriya et al., 2017). We found the aged naïve CD4 T cells showed reduced response to IL-6 (Brahmakshatriya et al., 2017). These APC also enhanced young responses, although to a lesser extent (Brahmakshatriya et al., 2017). This suggests that intrinsic reduction in IL-6 responsiveness with age causes many of the reduced naive CD4 responses and indicates a potential pathway to enhance aged responses. This helps explain why administering inflammatory cytokines enhanced aged CD4 T cell responses in earlier studies (Haynes et al., 2004). While there are likely to be other defects, higher dependence on IL-6 seems key (Devarajan et al., 2016). We suggest greater dependence on IL-6 production by APC is a key biomarker of age-associated unresponsiveness (Devarajan et al., 2016; Brahmakshatriya et al., 2017). During infection, PR signals to APC are responsible for inducing their ability to make high levels of IL-6 during subsequent cognate interaction (Jones et al., 2010), so this suggests higher levels of PR signals are needed in the aged. This caused us to consider whether aging “defects” in CD4 response may have developed to limit un-necessary response under circumstances without infectious microbes, while retaining responses to pathogens.

**FIGURE 1:**
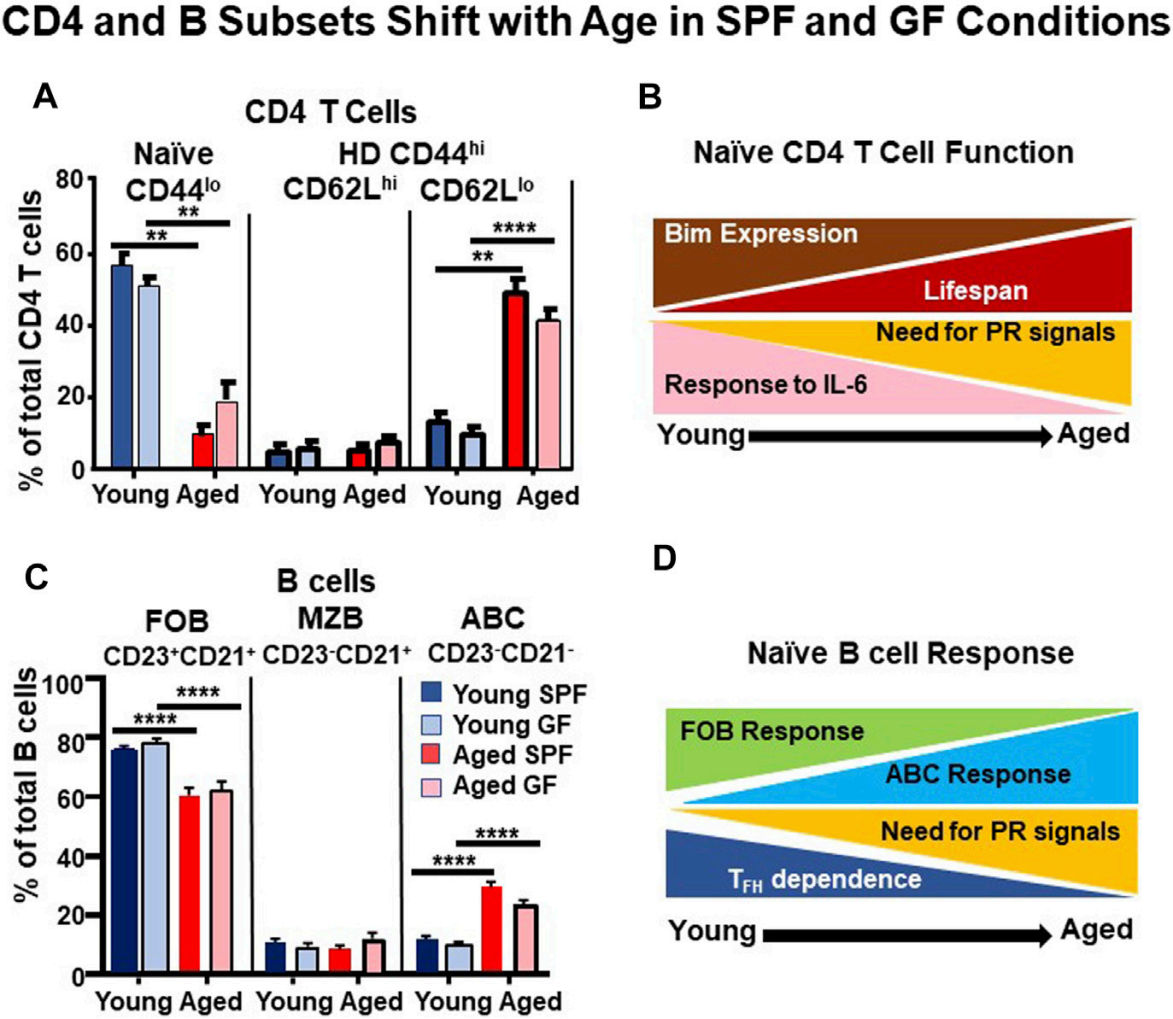
CD4 T and B Cell Subsets Shift with Age in SPF and GF Conditions. Spleen cells from unimmunized young (3–4 months) and aged (18–24 months) B6 female mice that were maintained in SPF vs GF conditions were analyzed by FACS. **(A)** We gated on resting CD4 T cells and analyzed for CD44 and CD62L expression to define: Naïve (CD44^lo^CD62L^hi^) and homeostatically differentiated (HD) CD44^hi^CD62L^hi^ and CD44^hi^CD62L^lo^ subsets. **(B)** A cartoon of changes in CD4 T cell responses. Aged naïve CD4 T cells are longer-lived, they express less BIM, respond less to IL-6, but depend more on pathogen recognition (PR) signals (Jones et al., 2010; Tsukamoto et al., 2010; Brahmakshatriya et al., 2017) **(C)** We gated on resting B cells (B220^+^CD19^+^) and quantified follicular B cells (FOB:CD23^+^CD21^+^), marginal zone B cells (MZB:CD23^-^CD21^+^), and aged-associated B cells (ABC:CD23^-^CD21^-^). **(D)** Cartoon of changes in B cell responses with age. The ABC can respond to infection and their response is highly dependent on PR signals, especially TLR7, while FOB respond less. The ABC depend less on T follicular cells (TFH) responses (Hao et al., 2011; Swain et al., 2017; Kugler-Umana et al., 2020). *n* = 9–12 pooled from 7–8 separate experiments. The statistical significance of combined experiments was determined by one-way ANOVA. Error bars represent the SEM: **p < 0.01; ****p < 0.0001.

IL-6 from APC is likely necessary at several stages of primary CD4 T cell response. We discovered a strict requirement for CD4 effectors to recognize antigen on activated APC again at the effector stage, which causes CD4 effectors to avoid programmed apoptosis and promotes transition into memory cells (Bautista et al., 2016). Indeed, adding activated APC at the effector phase enhances memory generation to inactivated influenza (Xia et al., 2020). Moreover, to drive further TFH effector responses, Ag on activated APCs needs to be present locally where the TFH reside (Devarajan et al., 2018; Devarajan et al., 2020). Ongoing infection is also necessary. Thus high systemic PR signals are needed both to activate the APC to drive TFH development and to induce yet-to-be determined innate pathways (Devarajan et al., 2018; Devarajan et al., 2020). While these studies are in young mice, we predict these PR signals will be of equal or greater importance in aged mice, due to the need for higher IL-6 levels to stimulate aged naïve T cells (Figure 1B).

As the naïve T cell population becomes smaller with age, the memory population, defined most often by high CD44 expression expands (Cicin-Sain et al., 2007; Kawabe et al., 2020). In free-living populations exposed to many sources of foreign antigens, including microbial pathogens and commensal organisms, it makes sense that conventional memory cells accumulate over time. Many memory CD4 and CD8 T cells are long-lived (Surh and Sprent, 2008) in comparison to naïve T cells and this would automatically drive their accumulation. Thus, it was initially surprising that T cells with a “memory phenotype” increase with age in unimmunized inbred laboratory animals kept in specific pathogen-free (SPF) conditions (Surh and Sprent, 2008; Chiu et al., 2013; Nikolich-Žugich, 2018). Moreover, CD8 and CD4 T cells of memory phenotype accumulate in germ-free (GF) mice not exposed to foreign or commensal microbes, and even in GF mice on elemental diets that lack food antigens (Pereira et al., 1986; Dobber et al., 1992; Haluszczak et al., 2009; White et al., 2017; Kawabe et al., 2020). The “memory phenotype” CD8 T cells express CD44, the “signature” memory cell marker, as well as elevated adhesion molecules (CD18, CD49d), activation-associated molecules (CD69), and survival cytokine receptors (CD122, CD132). Further studies show that distinct homeostatic cytokines IL-4 vs IL-15, which control T cell subset persistence (Sosinowski et al., 2013; White et al., 2017) act together with self-antigen recognition occurring in the thymus or periphery to drive the generation of these so-called “memory phenotype” CD44^+^CD8 T cells in unimmunized inbred mice. They likely develop and accumulate with age in humans as well (Su et al., 2013; White et al., 2016). Thus, there is a pathway for CD8 T cells to develop some memory cell characteristics independent of response to foreign or commensal Ag.

Different investigators have variously called the memory phenotype cells non-foreign antigen-derived memory (Kawabe et al., 2021), memory phenotype (Dobber et al., 1992; Haluszczak et al., 2009; White et al., 2017; Kawabe et al., 2020), innate memory (Kawabe et al., 2021), virtual memory (Akue et al., 2012; White et al., 2016) and yet others divide them into innate and virtual memory subsets (White et al., 2017). The CD44^+^ CD8 T cells have some of the positive attributes of true memory cells including more rapid cytokine production and response to some pathogens (Akue et al., 2012; Kawabe et al., 2020). However, since the CD44^+^ CD8 T cells develop in unimmunized mice in both SPF and GF housing conditions, using memory in their name contradicts the definition of classical foreign-Ag driven memory cells. Since they depend on homeostatic cytokines but not foreign Ag, we propose the term homeostatically-differentiated (HD) CD44^+^ cells is more accurate and hereafter refer to the non-foreign Ag-driven cells as HD CD44^+^ T cells (Figure 1).

Fewer studies have focused on the corresponding HD CD44^+^ population among CD4^+^ cells. Our own results shown in Figure 1A, support the findings of Kawabe et al. (2017), identifying equivalent proportions of CD4^+^ CD44^+^ CD62L^lo^ cells in spleens of young GF and SPF mice. The number of CD4 HD cells increases steadily as SPF mice age (Kawabe et al., 2017). We extend this observation and show the switch to a high and equivalent proportion of CD44 ^hi^ CD62L^lo^ CD4 T cells in aged GF and SPF mice and note there is also a small subset of CD44^hi^, CD62L^hi^ cells that do not increase with age (Figure 1A). There is thus a switch to a predominant population of HD CD44^+^ cells in the aged. Both CD44^+^ CD4 HD subsets also express somewhat upregulated CD49d, CD18, CD122 and slightly upregulated CD69, like their CD8 counterparts (Xia, Umana, Brahmakshatriya, Tonkonogy and Swain unpublished, not shown). The dramatic shift from naïve to HD cells in GF mice, in which antigen exposure is limited to food components, strongly suggests that the development of HD phenotype is an intrinsic, age-dependent program and we speculate it probably serves useful functions, such as providing rapid cytokine production in response to antigen. This supports the primacy of homeostasis in the development of HD CD44^hi^ cells and perhaps suggests this mechanism serves to replicate an alternate polyclonal pool that has some of the properties of memory cells. The function of these HD CD44 ^+^ cells in the aged needs to be evaluated in more detail to see if, like memory T cells, it is resistant to aging effects.

Figure 1B illustrates our working model of the suite of age-associated changes that occur equivalently in naïve CD4 T cells of SPF and GF mice. While we have only tested a few of the age-associated functions in GF mice so far, preliminary findings indicate the GF and SPF HD CD4 T cells develop a similar functional phenotype with age, including the key function of reduced response to IL-6 (Xia, Umana, Brahmakshatriya, Tonkonogy and Swain, unpublished).

### Age-Associated Changes in B Cell Function and B Cell Subsets

Conventional naïve B cell responses also become weaker with age (Frasca et al., 2008; Dorshkind and Swain, 2009; Scholz et al., 2013), compromising development of immunity to new antigens. Follicular B cells (FOB), the major naïve B cell population that responds to foreign antigen, decrease and undergo less somatic mutation in aged compared to young individuals (Frasca et al., 2008; Scholz et al., 2013). Additionally, most FOB responses depend on TFH, so the combined defects in FOB and TFH, make for highly impaired FOB antibody (Ab) responses. Other B cell subsets contribute to T-independent responses, including a small population of marginal zone B (MZB) cells that make short-lived, mostly IgM Ab responses but become less functional with age (Birjandi et al., 2011).

A recently described age-associated B cell (ABC) population, distinguished by lack of CD21 and CD23, accumulates with age (Hao et al., 2011; Rubtsov et al., 2011). We found that in untreated mice, many ABC have a naïve (sIgD^+^) phenotype and a small cohort responds to influenza A virus infection, differentiating into plasmablasts and plasma cells that produce influenza-specific Ab (Swain et al., 2017; Kugler-Umana et al., 2020). In fact, we find the immune response to influenza in aged mice is dominated by this T-independent ABC response and it depends heavily on viral recognition mediated by cytoplasmic toll like receptors (TLR) (Swain et al., 2017; Kugler-Umana et al., 2020). We are currently extending these studies to determine the potential of sIgD^+^ ABC cell precursors to contribute to protection against influenza.

ABC also develop in humans in response to infections (Nipper et al., 2018; Kugler-Umana et al., 2020) and they are found associated with autoimmunity (Rubtsov et al., 2013; Rubtsova et al., 2017). Many of the ABC studied in humans and autoimmune mice are non-resting B cells, with non-naïve phenotype, including the most widely studied ABC that express elevated T-Bet and CD11b or CD11c (Rubtsov et al., 2013; Rubtsova et al., 2017; Kugler-Umana et al., 2020). Whether these are derived from the putative naïve ABC that we have characterized is not clear, but all the ABC responses studied so far show strict dependence on TLR7 (Hao et al., 2011; Rubtsov et al., 2011; Rubtsov et al., 2013; Kugler-Umana et al., 2020). Other factors required for the development of the naïve ABC are not yet known, but our studies indicate ABC can respond to influenza without helper cells (Swain et al., 2017; Kugler-Umana et al., 2020). Importantly we find the naïve ABC population increases with age in GF as well as SPF mice (Figure 1C). We suggest that the shift in B cell phenotype with advancing age to one dominated by ABC is determined primarily by an intrinsic program that is homeostatically driven, and results in a highly PR-dependent response.

Thus, HD populations develop with advanced age among both naïve CD4 and B cell populations, supplanting much of the conventional naïve population with a unique surrogate population that responds mostly to infectious agents. We suggest these shifts are part of a program that protects against un-necessary responses.

## Discussion

Our studies point to a parallel strategy used in both CD4 T and B cells lineages that shift the requirements for the induction of immune responses as individuals age, so they require higher levels of pathogen recognition. We point out four major shifts that support this hypothesis.

First, there is marked reduction in conventional naïve CD4 T cells with age and the remaining naïve CD4 cells have a greater dependence on PR signals (Jones et al., 2010; Brahmakshatriya et al., 2017; Devarajan et al., 2016) (Figures 1A,B). Thymic involution is a primary cause, resulting in decreased naïve T cells. The remaining naïve CD4 T cells become longer-lived, and increased cellular age drives the development of age-associated changes in CD4 T cells (Tsukamoto et al., 2010). The remaining naïve CD4 T cells respond poorly to IL-6 signals needed for their expansion and differentiation to TFH, because they require higher levels of IL-6 produced by APC during their cognate interaction (Jones et al., 2010; Brahmakshatriya et al., 2017). Infection generates high persistent PR signals that license APC to make more IL-6 in subsequent interactions with T cells (Jones et al., 2010; Brahmakshatriya et al., 2017), and thus can restore some of the lost response (Xia et al., 2020).

Second, there is a striking increase of phenotypically distinct HD T cells expressing CD44 without CD62L (Figure 1A **)**. This impressive shift toward HD CD44^hi^ cells occurs in GF as well as in SPF mice (Figure 1A), suggesting that it is due to intrinsic programming (homeostasis), independent of extrinsic foreign or even commensal Ag.

Third, naïve FOB decrease and a unique ABC population accumulates as shown in Figure 1C, (Swain et al., 2017; Hao et al., 2011; Kugler-Umana et al., 2020). Lower generation of FOB cells and a reduction in their repertoire occurs with aging (Nikolich-Žugich, 2018; Frasca et al., 2008; Scholz et al., 2013). Additionally, due to the lack of TFH help and reduced responsiveness, FOB respond poorly to influenza (Lefebvre et al., 2016; Swain et al., 2017; Brahmakshatriya et al., 2017; Scholz et al., 2013). Instead, the ABC that we argue are naïve (Figures 1C,D) appear and respond to infection with influenza (Swain et al., 2017; Kugler-Umana et al., 2020). Further analysis of the signals regulating their responses are needed.

Fourth, in free-living conventional animals, memory CD4 T cells and B cells accumulate concurrently when driven by exposure to influenza and other infections early in life and drive naïve T and B cells to become Ag-specific long-lived memory cells that maintain their function and often persist throughout life (Haynes et al., 2003; Guerrettaz et al., 2008; Kogut et al., 2012; Devarajan and Swain, 2019). These provide strong protection to previously encountered strains. In addition, we show HD CD44^+^ T cells (aka innate memory, virtual memory, memory phenotype, or non-foreign Ag-driven) also accumulate, can rapidly make cytokines (Kawabe et al., 2017) and some may respond to pathogens. How they compare to bona fide memory cells in protective efficacy remains unclear and requires further study as does their ability to respond in aged mice and how they or conventional memory cells become autoimmune.

Why would the immune system evolve to have these properties? We suggest these age-driven transitions are an attempt to balance the need to respond robustly to dangerous pathogens, with the risk of generating potentially autoimmune cells, especially among resilient memory T and B cells. Humans encounter many non-threatening antigens and since memory cells persist and retain function with age, it seems prudent that the immune system would restrict their generation to infections with dangerous pathogens. Since the young can generate long-lived T and B memory cells that mount an effective response to recurrent pathogens, many of the necessary memory cells are present by middle age and it makes sense to increasingly limit new ones.

What are the consequences of aging-driven shifts on the capacity for successful immune responses? We speculate that in outbred, free-living mice and humans, responses are dominated by memory cells to previously encountered pathogens preserved into advanced age that largely retain function, along with a small pool of naïve CD4 T cells and ABC that respond only when abundant PR signals are present. Thus, most responses to already encountered pathogens will come from memory cells and will be effective. In contrast, responses to new antigens will be weak unless abundant PR signals are provided by infection or vaccines. In the elderly, the less effective responses can lead to dire consequences, as infection is cleared more slowly, and they are more frail and more susceptible to high mortality (McElhaney et al., 2016).

**FIGURE 2:**
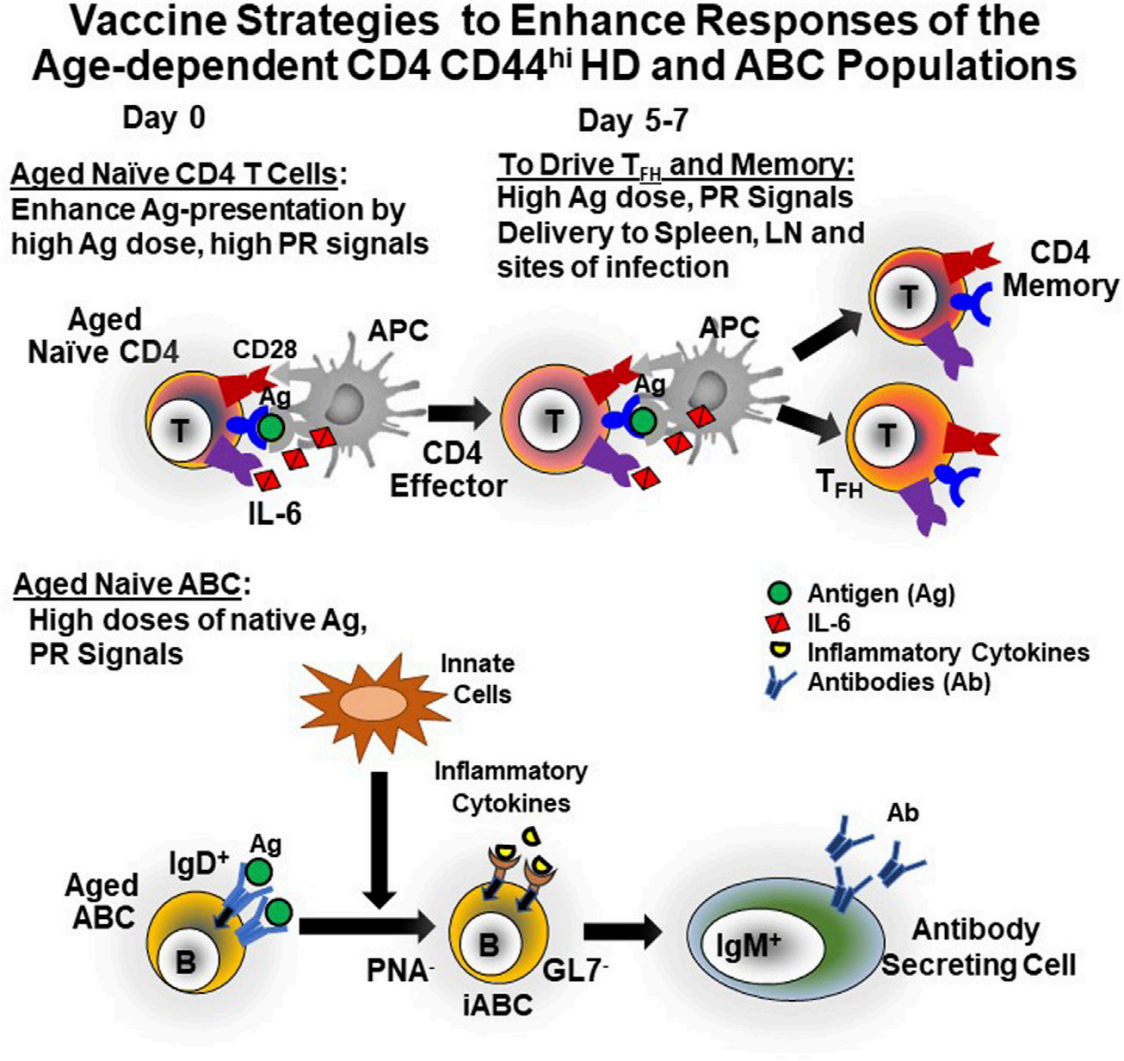
Strategies to Enhance Vaccines for the Aged. Our published studies suggest that in most current influenza vaccines both Ag and PR signals are limiting in quantity and of too short persistence, but the signals are present in abundance for at least a week with live influenza virus infection (Eaton et al., 2004; Haynes et al., 2004; Haynes et al., 2005; Tsukamoto et al., 2009; Tsukamoto et al., 2010; Devarajan et al., 2016; Brahmakshatriya et al., 2017; Xia et al., 2020). Therefore, we suggest that devising strategies to provide these both initially and at day 5 or 6 post vaccination will lead to increased generation of TFH and CD4 memory cells **(top)** and support generation of Ab-secreting cells from ABC **(bottom)**. Additionally, the TFH should enhance any residual FOB responses, leading to GCB, GC-derived Ab and memory B cells (not shown).

How can we modify vaccines to protect the aged against emerging pathogens? The findings that HD CD44^+^ CD4 cells and ABC are present in SPF, GF and foreign Ag-free mice clearly show that the maintenance of the immune system does not require activation by exogenous, foreign antigen. Instead, the development of different subpopulations is likely pre-programmed and dependent on self-recognition and constitutive homeostatic cytokines. Therefore, understanding how to harness these age-associated populations is critical, since they seem likely to be the major populations responsible for responses to new Ag in the elderly. The higher dependence of both naïve CD4 T cells and naïve ABC on PR signals, indicates that agonists that trigger downstream impacts of PR signals, need to be provided by vaccines that are designed to protect the aged against new threats such as new strains of influenza and emerging novel viruses such as SARS-CoV-2 as indicated in Figure 2.

We find that even in the young, development of CD4 memory and TFH cells depend on high levels of antigen presentation and PR signals that persist into the CD4 effector stage (Bautista et al., 2016; Devarajan et al., 2018; Devarajan et al., 2020; Xia et al., 2020) and on antigen presentation in sites of memory development (Devarajan et al., 2020). We predict the aged may need higher levels of these to induce protection. We therefore postulate that vaccines will require high levels of antigen and PR signals that persist for at least a week and that are provided in the necessary sites. If the aged require higher PR signals than the young, transient signals added repeatedly may be needed to reduce ill effects of extended inflammation (Lees et al., 2020). Vaccines to protect the elderly pose challenges because they will require such inflammation. Vaccines for recurrent pathogens that remain stable, could be given in middle age to build up conventional protective immunity before the age-associated changes are pronounced and frailty has developed (Lees et al., 2020). But when new pathogens or strains emerge like pandemic influenza, HIV and SARS-CoV2, it might be necessary to accept a certain level of inflammation to achieve strong immunity.

The COVID-19 pandemic revealed the remarkable vulnerability of older people exposed to emerging infections, with rates of death in the elderly several hundred-fold greater than in the young. It is heartening that the Pfizer-BioNTech and Moderna mRNA vaccines can effectively induce protective responses even in aged individuals (Anderson et al., 2020; Teo, 2021). We speculate that the mRNA – lipid nanoparticle vaccine platform provides the surrogates of pathogen infection that we believe are necessary for strong activation of even the aged immune system (Pardi and Weissman, 2020).

## Data Availability

The original contributions presented in the study are included in the article/supplementary material, further inquiries can be directed to the corresponding author.

## Abbreviations

ABC, age-associated B cells; DC, dendritic cells; FOB, follicular B cells; GF, germ-free; GC, germinal center; HA, hemagglutinin; HD, homeostatically-differentiated; iABC, infection-induced age-associated B cells; MZB, marginal zone B cells; PR, pathogen recognition; SPF, specific pathogen free; TFH, T follicular helper cells: TLR, Toll like receptor.
